# Wavelet-Like Transform to Optimize the Order of an Autoregressive Neural Network Model to Predict the Dissolved Gas Concentration in Power Transformer Oil from Sensor Data

**DOI:** 10.3390/s20092730

**Published:** 2020-05-11

**Authors:** Francisco Elânio Bezerra, Fernando André Zemuner Garcia, Silvio Ikuyo Nabeta, Gilberto Francisco Martha de Souza, Ivan Eduardo Chabu, Josemir Coelho Santos, Shigueru Nagao Junior, Fabio Henrique Pereira

**Affiliations:** 1 Industrial Engineering Graduate Program, Universidade Nove de Julho—UNINOVE, São Paulo 01525-000, Brazil; elanio@uni9.pro.br; 2 Informatics and Knowledge Management Graduate Program, Universidade Nove de Julho—UNINOVE, São Paulo 01525-000, Brazil; fernando.z.garcia@gmail.com; 3 Polytechnic School, Universidade de São Paulo—EPUSP, São Paulo 05508-010, Brazil; nabeta@pea.usp.br (S.I.N.); gfmsouza@usp.br (G.F.M.d.S.); ichabu@pea.usp.br (I.E.C.); josemir@pea.usp.br (J.C.S.); snjunior@usp.br (S.N.J.)

**Keywords:** dissolved gas analysis, power transformers, wavelet-like transform, autoregressive model

## Abstract

Dissolved gas analysis (DGA) is one of the most important methods to analyze fault in power transformers. In general, DGA is applied in monitoring systems based upon an autoregressive model; the current value of a time series is regressed on past values of the same series, as well as present and past values of some exogenous variables. The main difficulty is to decide the order of the autoregressive model; this means determining the number of past values to be used. This study proposes a wavelet-like transform to optimize the order of the variables in a nonlinear autoregressive neural network to predict the in oil dissolved gas concentration (DGC) from sensor data. Daubechies wavelets of different lengths are used to create representations with different time delays of ten DGC, which are then subjected to a procedure based on principal components analysis (PCA) and Pearson’s correlation to find out the order of an autoregressive model. The representations with optimal time delays for each DGC are applied as input in a multi-layer perceptron (MLP) network with backpropagation algorithm to predict the gas at the present and future times. This approach produces better results than choosing the same time delay for all inputs, as usual. The forecasts reached an average mean absolute percentage error (MAPE) of 5.763%, 1.525%, 1.831%, 2.869%, and 5.069% for C_2_H_2_, C_2_H_6_, C_2_H_4_, CH_4_, and H_2_, respectively.

## 1. Introduction

The transformer is one of the most important devices in the electricity distribution process, and reliable power distribution depends largely on the failure-free operation of this equipment. The failure of the transformer during operation can bring a significant loss of revenue to the utility, possible environmental damage, explosion and fire risks, and expensive costs of repair or replacement [1,2]. In the case in which these devices fail, operational life expectancy and reliability may change over the years and electricity to consumers may be interrupted. Therefore, the analysis of the condition and maintenance of the transformer are extremely important to ensure stable reliability of electricity [1,3,4,5].

When the power transformer is in normal operation, the insulating oil and solid insulating material will gradually deteriorate and a small amount of gas will be decomposed, including mainly hydrogen (H_2_), methane (CH_4_), acetylene (C_2_H_2_), ethylene (C_2_H_4_), ethane (C_2_H_6_), carbon monoxide (CO), and carbon dioxide (CO_2_). On condition of internal transformer failure occurring, the emergence speed of these gases is accelerated [6]. So, one of the most important tools for power transformer condition monitoring and internal fault diagnosis is the transformer oil gas chromatography test, known as dissolved gas analysis (DGA) [7,8,9,10].

Several studies have addressed the creation of power transformer condition monitoring systems based on DGA. Many techniques for predicting the concentration of gases have been proposed, such as wavelet least squares, support vector regression, neural network, deep learning, fuzzy model, and long short-term memory (LSTM), just to name a few.

In general, artificial intelligence techniques have been widely used to develop more accurate diagnostic tools based on DGA data [5,9,10,11,12,13,14]. In [9], for example, a new approach for diagnosing transformer failure was created based on gas rate and support vector machine (SVM). First, on the basis of the International Electrotechnical Commission Technical Committees (IEC-TC) 10 database, optimal dissolved gas rates are obtained by genetic algorithm designed for simultaneous DGA rate selection and SVM parameter optimization. In that work, three traditional methods were used: SVM DGA, backpropagation neural network (BPNN) DGA, and IEC criteria, and three-key IEC gas proportions with SVM and back propagation neural network were employed to compare accuracy. The SVM technique also served as a basis for the approaches in [11,13,15]. The authors in [13] have used the least squares support vector machine (LS-SVM) for dissolved gases forecasting (H_2_, CH_4_, C_2_H_2_, C_2_H_4_, and C_2_H_6_) and assessing incipient faults of transformer polymer insulation. Meanwhile, in [15], a new approach has been proposed to combine technical wavelet regression with LS-SVM for the prediction of dissolved gases in power transformers immersed in oil. In [10], the authors have used a fuzzy inference system (FIS) to determine absolute concentrations of free and dissolved transformer oil, total dissolved combustible gases, total combustible gases, proportions of some gases with each other, and gas rates increasing to detect the decomposition of transformer isolation papers. A similar approach has been proposed in [5], in which an adaptive neuro fuzzy inference (ANFIS) system was employed to estimate the transformer isolation degradation rate with the input variables H_2_ (hydrogen), CH_4_ (methane), N_2_ (nitrogen), O_2_ (oxygen), CO (carbon monoxide), CO_2_ (carbon dioxide), C_2_H_6_ (ethane), C_2_H_4_ (ethylene), C_2_H_2_ (acetylene), and TDCG (total dissolved combustible gas).

In general, these numerous studies have used artificial intelligence techniques as regression to predict gas concentration or faults in power transformers. More specifically, the use of prediction models in connection with the wavelet transformed has been addressed in some recent works to improve the forecast [13,14,15]. Despite satisfactory results, those approaches may not be the most efficient in predicting future values of the variable of interest, especially for a multi-step ahead forecast. Several empirical studies show that learning long-term time dependencies can be difficult for gradient-descent algorithms, which are more effective, converge faster, and generalize better in nonlinear autoregressive neural network models than in other neural networks [14,16,17,18,19,20]. Autoregressive models based upon neural networks specify that the output variable depends, in a non-linear way, on its own past values and on a stochastic imperfectly predictable term. Thus, the prediction of future values of the output variable can be realized from its past and present values. Additionally, the prediction model can also consider present and past values of one or more auxiliary external variables, resulting in a nonlinear autoregressive model with exogenous variables.

In this sense, the authors in [14] proposed a combination of a nonlinear autoregressive neural network model with the discrete wavelet transform, resulting in a high-accuracy multi-step ahead forecast of in-oil gas concentrations. The authors investigated the use of different wavelet functions and different time delays in the autoregression model, but they did not assess how different delays in external series can influence the values of the output series.

In fact, the definition of the optimal input and output delays is one of the main limitations of an autoregressive model. In general, in multidimensional models with *n* external variables, equal variables delays are adopted. This means that the prediction of the output value at time *t* + 1, *y*(*t* + 1), is performed using the past outputs *y*(*t*), *y*(*t* − 1),…, *y*(*t* − *d_y_*) and the past observations *u_i_*(*t*), *u_i_*(*t* − 1),…, *u_i_*(*t* − *d_u_*) of the external variables *u_i_* as inputs, *i* = 1,…,*n*. In addition, the adoption of many inputs can increase the complexity of the forecasting model and reduce its accuracy. Thus, some difficulties and limitations remain despite the advances, motivating research for new models to be conducted.

The investigation of the use of different time delays in external series that influence the output does not seem to have received the necessary attention, especially considering that there is a strong correlation between the concentrations of different gases and failures in transformers. This work seeks to contribute to overcome this limitation by proposing a wavelet-like transform to optimize the order of the factors in an autoregressive neural network model, with some exogenous variables, to predict the dissolved gas concentration in power transformer oil.

The main objective of this work is to determine the optimal delay for each input and for the output to create an autoregressive model with a reduced number of inputs and with competitive precision in relation to the literature. The hypothesis is that wavelet-like approximations of the external variables and the output variable incorporate the temporal memory of the autoregressive model. In addition, the selection of the best approximation for each variable determines the ideal delay for each input while reducing the size of the model, as each sample of the approximation is calculated considering a time window of the series.

Consequently, the contributions of the proposed approach can be stated as follows:

1. Development of an approach based on a wavelet-like transform that determines the optimal delay for each external variable and for the output variable in an autoregressive prediction model;
2. A prediction model with high precision as it focuses on the trend of the input signals from the noise-free approximations calculated by the wavelet transform;
3. Expansion of knowledge of the temporal relationship between gases underlying degradation process of the insulating oil and solid insulating material;
4. Reduction of the number of input variables in the autoregression model when using the approximations resulting from transformations with wavelets of different lengths, which already consider the time delay determined for each variable.

The remainder of this paper is organized as follows. The related theory is discussed in Section 2—dissolved gas-in-oil analysis in Section 2.1, discrete wavelet transform in Section 2.2, and nonlinear autoregressive exogenous model in Section 2.3. In Section 3, materials and methods will be presented, followed by the results in Section 4, discussion in Section 5, and finally the conclusion in Section 6.

## 2. Related Theory

### 2.1. Dissolved Gas-In-Oil Analysis

Power transformers are one of the most important devices for the electrical system, and more than 90% of transformers are immersed in oil [21]. Dissolved gas-in-oil (DGO) is a simple parameter used to monitor energized power transformers and assess the condition of power transformers (PTE) [22,23,24].

Because of the thermal and electrical stresses experienced by the insulation of operating transformers, paper and oil decomposition occurs, generating gases that dissolve in the oil and reduce its dielectric strength. Thus, concentrations of various gases dissolved in the transformer oil owing to the decomposition of the oil and paper insulation [22].

Depending on the composition and the level of gas concentration, the power transformer may fail. Usually, the gases that are present as dissolved gases in insulating oil in transformers are hydrogen (H_2_), methane (CH_4_), ethane (C_2_H_6_), ethylene (C_2_H_4_), acetylene (C_2_H_2_), carbon dioxide (CO_2_), carbon mono-oxide (CO), oxygen (O_2_), and nitrogen (N_2_) [1].

There are a couple of DGA-based interpretive methods for detecting power transformer failure that use a relation between two gases to determine a possible problem. The methods are gas key; IEC ratios; the graphical representation, IEC 60599, Duval; and Doernenburg, Rogers, among others [4,11,21]. As an example, in the following, we show three of these methods and their respective gas concentration levels. Table 1 shows the fault description for gas concentration, problem description, and normal and abnormal values for each gas concentration. In Table 2, we have fault diagnosis by the Dornenburg ratio method (R1, R2, R3, R4) and fault type, while Table 3 shows fault classification using IEC ratio codes (C_2_H_2_/C_2_H_4_, CH_4_/H_2_, C_2_H_4_/C_2_H_6_) and fault type [2,24].

### 2.2. Discrete Wavelet Transform

Wavelet transform (WT) is a widespread signal processing technique. In the last decades, several algorithms of compactly supported wavelet have been created by mathematical analysis and signal processing communities. In fact, several works attempt to motivate and explain the basic ideas behind wavelets, what makes them so successful in many applications in different areas, as well as some limitations [25,26,27,28].

Mostly, WT is used for trend analysis, correlation and coherence between two time series, cross-spectral analysis, and space-based verification wavelets, while there are limited applications related to forecasting [29].

Discrete wavelet transform (DWT) is any wavelet transformation for which wavelets are discretely sampled, meaning this is a discrete set of the wavelet scales and translations [29]. The basic idea of this technique is to exploit the correlation present in most real-life signals to build a sparse approximation [14]. So, DWT possesses many favorable properties that are useful for researchers in the time series data mining field [1].

The dependency on discrete one-dimensional wavelet transform is presented in Equations (1)−(3).

(1) $$f\left(t\right)=\sum_{k}A_{m,k}\varphi_{m,k}\left(t\right)+\sum_{j=1}^{m}\sum_{k}D_{j,k}\psi_{j,k}\left(t\right)$$

(2) $$H\left(\omega \right)=\sum_{n\in Z}h_{n}e^{-in\omega }$$

(3) $$G\left(\omega \right)=\sum_{n\in Z}g_{n}e^{-in\omega }$$

where $H\left(\omega \right)$ is the transfer function of high-pass filter, $G\left(\omega \right)$ is the transfer function of low-pass filter, and they filter the signs of low and high frequency keeping important information from the original signal. $h_{n},g_{n}$ are the coefficients depending on the mother wavelet; $A_{m,k}$ is an approximated profile; $D_{j,k}\;$ is the detail profile; j,m are the decomposition level of the wavelet transform; $\psi_{j,k}\left(t\right)$ is mother wavelet and $\varphi_{m,k}\left(t\right)\;$ is the scaling function.

Daubechies wavelets (DW), *dbN*, offer a family of orthogonal transformations, where *N* refers to the number of vanishing moments, which generally vary from *db2* to *db22* and have the ability to accurately approximate constant and linear functions and a relatively simple form [1,27].

### 2.3. Nonlinear Autoregressive Exogenous Model

A time series is any set of observations organized in time; usually measurements are made at evenly spaced times, for example, daily pollution values, monthly temperature values, daily values of electricity consumption, and daily stock exchange indices, among others [30].

An exogenous nonlinear autoregressive model (NARX) in time series modelling is a nonlinear model that has exogenous inputs, in which the model relates past values of the same series and current and past values of the driving series (exogenous), that is, externally determined series that influence the series of interest, as defined in Equation (4) for *l* exogenous variables:

(4) $$y_{t}{=F(y}_{t-1}{,\;y}_{t-2}{,\ldots ,\;y}_{t-d_{y}}{,\ldots ,\;u}_{1,t}{,\;u}_{1,t-1}{,\ldots ,\;u}_{1,t-d_{u1}}{,\ldots ,\;u}_{l,t}{,\;u}_{l,t-1}{,\ldots ,\;u}_{l,t-d_{ul}})+\varepsilon$$

where *y* is the output variable; *u_i_*, *i* = 1,…,*l* are externally determined variables; *d_ui_* is the order of variable *u_i_*; *ε* is the error term; and *F* is some non-linear function, such as a polynomial for example.

There is a trend toward the adoption of computational techniques and many effective attempts have been developed, such as the following: the authors of [14] have used NARX to predict gas concentration in power transformer oil. Meanwhile, the authors of [5] and [10] have created an autoregressive model using ANFIS models to detect and isolate, as well as perform transformer paper expected life estimation.

As can be seen, there are a couple of autoregressive models that have been used to assist in the prediction of DGA and the health status of the power transformer [7]. However, in all the researched papers, the order of the factors is always the same, that is, $d_{y}=d_{u1}=\cdots =d_{ul}$ in Equation (4).

## 3. Materials and Methods

The proposed approach relies on a wavelet-like transform to optimize the order of the factor (gas concentrations) in a nonlinear autoregressive model with exogenous variables. This means to define the optimal order $d_{y},d_{u1},\cdots ,d_{ul}$ for each gas concentration.

**Figure 1:**
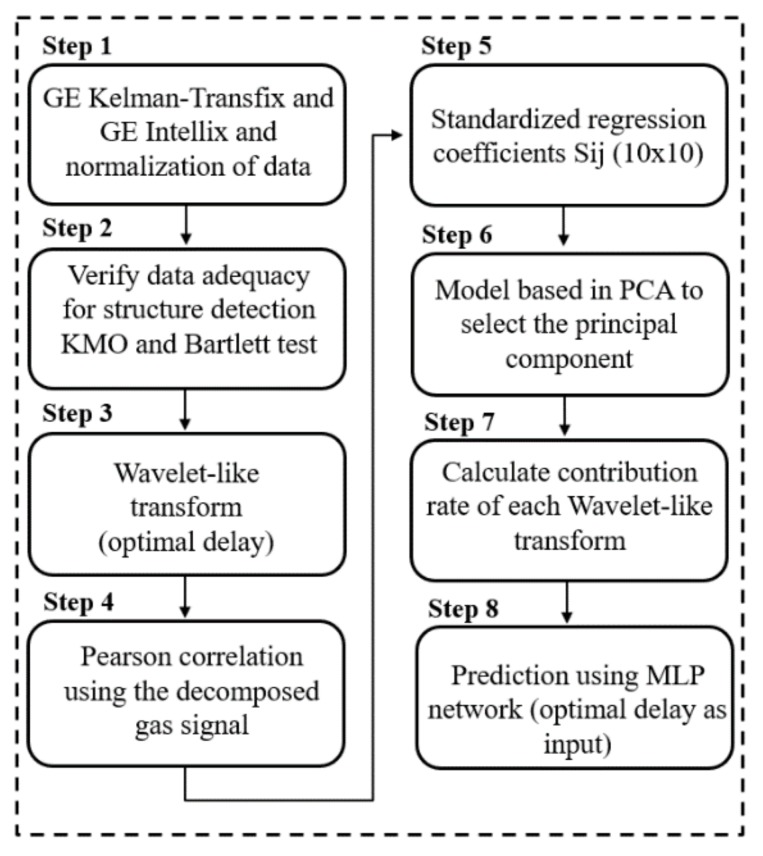
Model for selection and contribution rate of gases concentration and prediction. MLP, multi-layer perceptron; PCA, principal components analysis; KMO, Kaiser–Meyer–Olkin; GE, General Electric.

Thus, the approach proposed has the following steps: step 1, gas concentration acquisition and data normalization; step 2, Kaiser–Meyer–Olkin (KMO) and Bartlett test; step 3, wavelet-like decomposition of gas concentration; step 4, Pearson’s correlation; step 5, standardized regression coefficients; step 6, a model using principal components analysis (PCA) to select the principal component; step 7, calculation of contribution rate for each wavelet decomposition level; and, finally, step 8, prediction using the best time delay as input in a multi-layer perceptron (MLP) network. All these steps are illustrated in Figure 1 and described in detail as follows.

***Step 1:***

Usually, interpretation techniques such as Duval triangle are applied to the information on the concentration of gases in the transformer oil, which is collected using an equipment such as Morgan Calisto, Luman Sense Smart DGA, General Electric (GE) Transfix, Qualitrol DGA 150, or others [8].

Initially, this work collected a set of 190 historical oil-dissolved gas data from a transformer equipped with a GE Kelman-Transfix (GE—General Electric, Sao Paulo, Brazil) and GE Intellix BMT 330 (GE—General Electric, Sao Paulo, Brazil). In this stage, the variables pointed out by [10,11,12,13] are C_2_H_2_, C_2_H_4_, C2H_6_, CO, CO_2_, CH_4_, O_2_, and H_2_. However, H_2_O and combined gas concentrations were added as input, resulting in ten variables. Before the next step, all the data were normalized between 0 and 1.

***Step 2:***

The KMO test is applied to verify the measure adequacy sampling for each variable in the model [31] and the Bartlett test to test the hypothesis that the correlation matrix is an identity matrix, which would indicate that variables are unrelated, and thus unsuitable for structure detection [32].

KMO (1977) is a criterion for identifying whether a factor analysis model being used is adequately fitted to the data, testing the overall consistency of the data [31]. Meanwhile, Bartlett’s sphericity test is a technique created by Maurice Stevenson Bartlett in 1937, which indicates the strength of the relationship between variables.

***Step 3:***

At this stage, DWT is used in two forms. In the first one, each gas concentration is decomposed keeping level of decomposition in 1 while changing the wavelet from *db2, db4*,…, to *db20*, in order to create smooth approximations of the original gas concentration using the low frequency filters. Additionally, the wavelet transform is applied in the gas concentrations in reverse chronological order so that each sample of the approximation is created with values passed from the original signal.

Considering *m* samples from a time series in reverse chronological order, that is, the most recent samples at the beginning, $S=\left(s_{t},\;s_{t-1},\cdots ,s_{t-k},\cdots ,s_{t-m+k},\cdots ,s_{t-m+2},s_{t-m+1}\right)$, and a low pass wavelet filter H of length k, $H=\left(h_{0},\;h_{1},\;\cdots ,\;h_{k-1}\right),\;k\ll m$, Equation (5) defines the application of the transform to the signal S to create an approximation $S_{dbk}^{’}=(s_{t}^{’},\;s_{t-1}^{’},\;\cdots ,\;s_{t-\left(m/2\right)+1}^{’})$ with time delay k−1, as proposed in this work,

(5) $$s_{t-j}^{’}=\overset{k-1}{\underset{i=0}{\sum }}h_{i}s_{t-i-\mathrm{jk}},\;\;j=0,\ldots ,\frac{m}{2}-1$$

Approximations $S_{\mathrm{dbk}}^{’}$, k=2, 4,…, 20, with half the length of the original signal, m/2, for each Daubechies wavelets from *db2* to *db20*, are created, resulting in 10 approximations for each time series S. Here, we have 190 samples of each gas concentration.

***Step 4:***

Unlike the authors of [33], who have used Pearson’s correlation coefficient between the constant characteristic parameter and the candidate of the variable characteristic parameters to verify the concentration of gas that presents the best correlation to electrical faults, this work uses the Pearson’s correlation to calculate a relationship between the various approximations created for gas concentrations with different time delays (wavelets of different lengths). Thus, this step results in a matrix X with 110 columns and 190 rows, such that the 110 columns represent the time *t*, *t* − 2, through *t* − 20 of each gas concentration, which generates 110 input variables.

***Steps 5 and 6:***

In these steps, we apply PCA in the matrix *A* created from the relation between inputs Xj (gas concentrations delayed at time *t* − 2 to *t* − 20 according to wavelet-like transform) and output Yi (a gas concentration in time instant *t*). So, the values of *A* are calculated as standardized regression coefficients *a_ij_* (Equation (6)) for each input and output, describing the relationship between the concentration of a given gas and the approximations created for all other gases in different time delays generated by the wavelet transform. Therefore, a square matrix is created for each gas concentration, in which the PCA is applied to select the main components that represent at least 99% of the original data variation, generating a supervised PCA (SPCA), according to [34,35].

(6) $$a_{ij}=\frac{X_{j}^{T}Y_{i}}{\sqrt{X_{j}^{T}Y_{i}}}$$

***Step 7:***

The contribution of each time delay is calculated as follows: $C_{i}\left(j\right)=\sum_{i=1}^{p}\left(A_{j}^{T}A_{j}^{’}\right)\lambda_{i}$, in which *A* represents the input data, *λ* are corresponding eigenvalues, *A*’ is the representation of *A* in the principal component space, and *p* is the most important principal component [35].

***Step 8:***

An MLP neural network is trained with the Levenberg–Marquardt backpropagation algorithm with 100 epochs, 1 input layer, 1 hidden layer, and 1 output layer. The neurons in the hidden layer were used following two approaches—the first one following [36], which propose a method using $N_{h}{=2}^{n}–\;1$, and the second following [37], proposing $N_{h}{=(4n}^{2}+3)/{(n}^{2}\;–\;8)$, where $N_{h}$ corresponds to the best neurons numbers and n is the number of input parameters.

Unlike [3], we normalize the input data between –1 and 1 for applying a population-based metaheuristic algorithm to optimize the structure of the MLP neural network with back propagation algorithm. We propose using the optimal time delays made with the wavelet as input in an MLP with a backpropagation algorithm.

In order to test the temporal relationship between gases underlying the degradation process of the insulating oil and solid insulating material, five gas concentrations were chosen, as the main methods basically used by the IEC ratios and Rogers and Dornenburg ratios are C_2_H_2_, C_2_H_4_, C_2_H_6_, CH_4_, and H_2_ to identify possible power transformer faults.

**Figure 2:**
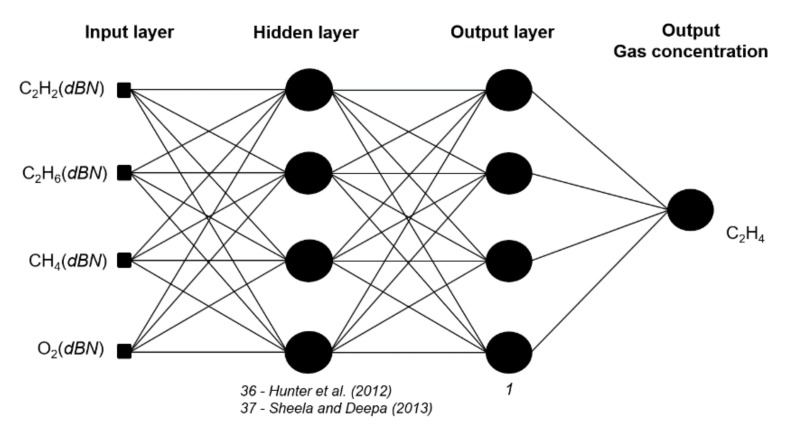
Gas concentration C_2_H_4_ decomposed by Wavelet *db2* to *db20.*

Figure 2 shows an example of the neural network architecture to predict gas concentration C_2_H_4_, where the optimal time delays for C_2_H_2_, C_2_H_6_, CH_4_, and O_2_ are selected according Pearson’s correlation and PCA.

Regarding the output, 95 samples related to odd days were selected to create the matrix *A*, as well as to train and test the forecasting model, as it is necessary to put the input and output data with the same length.

Similar experiments were carried out for the other gases: when the output is C_2_H_2_, the inputs are the approximations of C_2_H_4_, C_2_H_6_, CH_4_, and O_2_ with their respective optimal delays defined by the proposed approach; when the output is C_2_H_6_, the inputs are the wavelet approximations of C_2_H_2_, C_2_H_4_, CH_4_, and O_2_; and so on.

## 4. Results

Firstly, we evaluated the results of the KMO and Bartlett test. In Table 4, the KMO test indicated 0.743, while at the same time, the Bartlett test indicated 0; for this reason, these data are suitable for data structure detection, while the Bartlett test indicates that a factor analysis may be useful with your data.

The next stage shows the resulting of selection and contribution rate of decomposition of each variable. Table 5 shows the contribution rate per gases concentration (normalized).

Each variable presents a different importance rate and first order, C_2_H_6_ has Wavelet *db20*, which means that all gas concentration have to delay in time instant *t* − 20, while *db8* has more impact in CH_4_, *db18* in O_2_, and so on (see Table 5).

The level of correlation of the time delays for each gas concentration in is shown Table 6, wherein the values marked in bold and italics are the time delays that have higher correlation with C_2_H_2_, C_2_H_6_, C_2_H_4_, H_2_, and CH_4_.

The following shows the results of the neural network prediction for two gases concentrations using 8 and 15 neurons in the hidden layer, as the methods DGA IEC ratios, as well as the Rogers and Dornenburg ratios, basically use the following to analyze the potential problems in power transformers: CH_4_ gas, H_2_, C_2_H_2_, C_2_H_4_, and C_2_H_6_.

**Figure 3:**
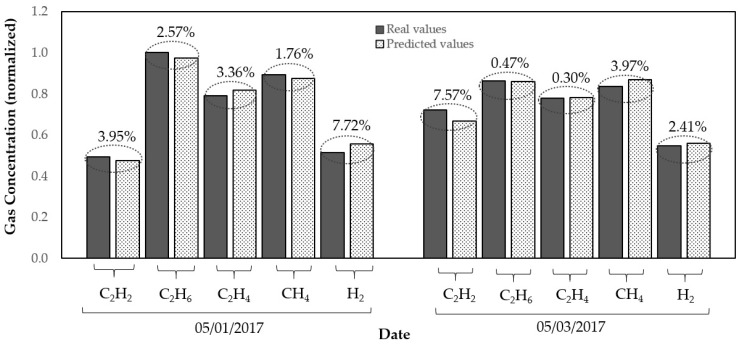
Real and predicted values of the gas concentration for two days.

The results presented in Table 7 show us an average MAPE for two days of 1.525% for C_2_H_6_ and 1.831% for C_2_H_4_. Meanwhile, Figure 3 compares the predicted values with the real values for the five gas concentrations. As can be seen, the selection of the optimal time delay in gas concentration can improve prediction accuracy, when comparing predictions with the input variables at the same time t − 2 and t − 4 (Table 7).

## 5. Discussion

This study aimed to study the optimal time delay of each gas concentration impacting the gases H_2_, CH_4_, C_2_H_2_, C_2_H_4_, and C_2_H_6_ (Table 6 and Table 7), in which a DGA technique subsequently be used to detect the defect in the power transformer.

The approach using a wavelet-like transform and SPCA shows the contribution rate of different time delays of each gas concentration, which differs from the proposal of recent works, such as, for example [13,14]. In [14], for example, despite testing different wavelet functions and different delays, all models adopted the same time delay for external variables. Here, the approach shows the rate and order of importance and wavelet-like order for ten gas concentrations (Table 5), indicating that *db20* (*t* − 20), *db8* (*t* − 8), and *db18* (*t* − 18) are the three most important time delays for the gas concentrations C_2_H_6_, CH_4_, and O_2_, respectively. This result shows that the effect that a given gas suffers from other gases varies differently over time for each gas.

We have used Pearson’ s correlation to consideration the impact of each time delay as using different time delays *t* − 2 to *t* − 20 in each gas concentration, showing, for example, that to predict the concentrations of C_2_H_2_, the best time delays for the other gas concentrations are as follows: *t* − 12 for C_2_H_4_, *t* − 6 for C_2_H_6_, *t* − 10 for CH_4_, and *t* − 8 for H_2_. It is important to highlight that a traditional autoregressive model that adopts the same delay for all variables would not have identified this relationship. In addition, this is a very important result for calibrating monitoring systems, as it indicates that any variation in C_2_H_4_, for example, will take about 12 units of time to reflect on the concentration of C_2_H_2_. A similar analysis applies to other gases.

A similar kind of relationship of different gases has been studied in [38] and [33]. In [38], the authors have studied a correlation between the five gas concentrations, by applying the value of grey relational grade to reveal the relationships between gas features. Those authors show that the grey relation analysis is efficient in selecting and removing redundant features from the set of input variables. However, it does not consider any time delay in sampling the input series of gas concentrations. On the other hand, the authors in [33] have used correlation coefficients of gas concentration CO as a constant characteristic parameter for the correlation of time series analysis and H_2_, CH_4_, C_2_H_2_, C_2_H_4_, and C_2_H_6_ as characteristic variable parameters to be used to distinguish electrical faults from thermal faults.

However, approaches based on autoregressive models apply the same order for all input variables and do not take into account the time delay relationship between gas concentrations. Notwithstanding, we have seen that the optimal selection of the time delay for each concentration of gas affects the output.

Regarding forecast accuracy, this approach shows some better predictions than [33,38,39] (see Table 8).

It is important to highlight the low computational cost of the proposed model, because it takes a matter of seconds to run. In the example above regarding the prediction of the C_2_H_2_ concentration, instead of input 12, 6, 10, and 8 passed values of the gases C_2_H_4_, C_2_H_6_, CH_4_, and H_2_, respectively; according to in Equation (4), we simply use the corresponding four approximations created by the wavelet-like transform for each exogenous gas.

## 6. Conclusions

This work presented an approach based on the wavelet transform to determine the ideal time delay for each gas concentration and use it as a regression model in an MLP network. The application of a wavelet-like transform creates sparse approximations of gas concentrations with different time delays, and enables us to define an accurate and computationally efficient prediction model. The prediction model has high precision as it focuses on the trend of the input signals from the noise-free approximations calculated by the wavelet transform. Moreover, it reduces of the number of input variables in the autoregression model when using the approximations resulting from transformations with wavelets of different lengths, which already consider the time delay determined for each variable.

In addition to simply assessing the relationship of different gases, the proposal makes it possible to identify the relationship between a specific gas and delayed approximations of all other gases. This is a fundamental result for monitoring systems, as it indicates that variations in different gases take different times to be reflected in the concentrations of other gases under observation. As an example, it was observed that variations in ethane affect acetylene concentrations more quickly than variations in ethylene. So, the approach extends the discussion of the temporal relation between gas concentrations, providing an expansion of knowledge of the temporal relationship between gases underlying degradation process of the insulating oil and solid insulating material.

Thus, wavelet-like approximations of the external variables were an effective tool to incorporate temporal memory of the autoregressive model and to determine the ideal delay for each input. As future research, we suggest investigating other feature selection techniques to evaluate the results of Pearson’s correlation and PCA.

## Figures and Tables

**Table 1:** Fault description for gas concentration.

Chemical Formula	Normal	Abnormal	Problem Description
H_2_ (hydrogen)	<150 ppm	>1000 ppm	Electric discharge (corona effect, low partial discharge)
CH_4_ (methane)	<25 ppm	>80 ppm	Secondary indicator of an arc or serious overheating
N_2_ (nitrogen)	1%–10%	NA	-
O_2_ (oxygen)	0.03%	>0.5%	Transformer seal fault
CO (carbon monoxide)	<500 ppm	>1000 ppm	Cellulose decomposition
CO_2_ (carbon dioxide)	<10,000 ppm	>15,000 ppm	Cellulose decomposition
C_2_H_6_ (ethane)	<10 ppm	>35 ppm	Secondary indicator of thermal fault
C_2_H_4_ (ethylene)	<20 ppm	>100 ppm	Thermal fault (overheating local)
C_2_H_2_ (acetylene)	<15 ppm	>70 ppm	Electric fault (arc, spark)

**Table 2:** Fault diagnosis by the Dornenburg ratio method.

Ratio R1 (CH_4_/H_2_)	Ratio R2 (C_2_H_2_/C_2_H_4_)	Ratio R3 (C_2_H_2_/CH_4_)	Ratio R4 (C_2_H_6_/C_2_H_2_)	Fault Type
>1	<0.75	<0.3	>0.4	Thermal decomposition
<0.1	Insignificant	<0.3	>0.4	Corona
>0.1 and <1	>0.75	>0.3	<0.4	Arcing

**Table 3:** Fault classification using International Electrotechnical Commission (IEC) ratio codes.

C_2_H_2_/C_2_H_4_	CH_4_/H_2_	C_2_H_4_/C_2_H_6_	Fault Type
0	0	0	Normal aging, no fault
Insignificant	1	0	Partial discharge of low energy density
1	1	0	partial discharge of high energy density
1	0	1	Discharges of low energy
1	0	2	Discharges of high energy
0	0	1	Thermal fault of <150 °C
0	2	0	Thermal fault of ≥150 °C and ≤300 °C
0	2	1	Thermal fault of >300 °C and ≤700 °C
0	2	2	Thermal fault of >700 °C

**Table 4:** Kaiser–Meyer–Olkin (KMO) and Bartlett sphericity test.

KMO and Bartlett Test		
KMO sampling adequacy measure		0.743
Bartlett’s sphericity test	Aprox. Square-Qui	418.644
	Gl	45
	Sig.	0

**Table 5:** Importance order and rate of each wavelet order for gas concentration.

Importance Order	Wavelet-Like Order	Gas	Importance Rate	Importance Order	Wavelet Order	Gas	Importance Rate
1	*db20*	C_2_H_6_	1.000	11	*db4*	GC	0.636
2	*db8*	CH_4_	0.858	12	*db20*	H_2_	0.581
3	*db18*	O_2_	0.848	13	*db8*	C_2_H_6_	0.575
4	*db20*	CH_4_	0.803	14	*db8*	H_2_	0.572
5	*db16*	O_2_	0.793	15	*db20*	C_2_H_4_	0.568
6	*db12*	CO_2_	0.791	16	*db20*	H_2_O	0.539
7	*db10*	CO_2_	0.779	17	*db16*	GC	0.53
8	*db6*	CH_4_	0.776	18	*db18*	GC	0.529
9	*db14*	O_2_	0.692	19	*db14*	H_2_	0.507
10	*db2*	GC	0.644	20	*db20*	CO	0.495

**Table 6:** Correlation level of time delays.

Gas Concentration (Delayed)	C_2_H_2_	C_2_H_6_	C_2_H_4_	H_2_	CH_4_
C_2_H_2_*(db2)*	0.01254	0.00116	***0.01904***	0.00029	0.00608
C_2_H_2_*(db4)*	0.00449	0.00116	0.01796	0.00109	***0.00689***
C_2_H_2_*(db6)*	0.00000	0.00032	0.01232	***0.00548***	0.00490
C_2_H_2_*(db20)*	***0.01769***	*0.00563*	0.00000	0.00137	0.00029
C_2_H_6_*(db4)*	0.01103	0.01440	0.03349	0.01061	***0.00314***
C_2_H_6_*(db6)*	***0.02190***	***0.02045***	***0.04203***	***0.01232***	0.00281
C_2_H_4_*(db8)*	0.00922	0.00017	0.00073	***0.00292***	0.00026
C_2_H_4_*(db10)*	0.00865	0.00003	0.00130	0.00044	0.00044
C_2_H_4_*(db12)*	***0.01082***	0.00010	***0.00240***	0.00000	***0.00185***
C_2_H_4_*(db20)*	0.00706	***0.00144***	0.00130	0.00036	0.00010
H_2_*(db2)*	0.00410	0.00130	0.00410	***0.00504***	0.00102
H_2_*(db6)*	0.00068	***0.00348***	0.00384	0.00026	0.00109
H_2_*(db8)*	***0.00130***	0.00250	0.00240	0.00048	0.00017
H_2_*(db20)*	0.01300	0.00130	***0.00608***	0.00176	***0.00270***
CH_4_*(db4)*	0.00005	0.00281	0.00036	***0.00336***	0.00044
CH_4_*(db10)*	***0.00922***	0.00058	0.00032	0.00023	0.00144
CH_4_*(db14)*	0.00044	0.00000	0.00006	0.00012	***0.00281***
CH_4_*(db20)*	0.00336	***0.00336***	***0.00212***	0.00160	0.00020

**Table 7:** Predicted values with and without selection of best delay time. MAPE, mean absolute percentage error.

	Gas Concentration C_2_H_6_				Gas Concentration C_2_H_4_		
Number of Neurons	Inputs/Date	05/01/2017	05/03/2017	Average MAPE%	05/01/2017	05/03/2017	Average MAPE%
	Real	1	0.864	-	0.791	0.779	-
8 neurons	Selection of time delay	0.972	0.756	7.645	0.789	0.657	7.891
	t − 2	0.842	0.849	8.811	0.749	0.753	4.308
	t − 4	0.769	0.889	13	0.786	0.847	4.64
15 neurons	Selection of time delay	0.974	0.86	***1.525***	0.817	0.781	***1.831***
	t − 2	0.818	0.919	12.294	0.794	0.805	1.909
	t − 4	0.83	0.864	8.492	0.65	0.995	22.777

**Table 8:** Comparison of predicted gas concentrations.

Average MAPE(%)						
Authors	Approach	C_2_H_2_	C_2_H_4_	C_2_H_6_	CH_4_	H_2_
Wang et al., 2015	Time series correlation	38.900	42.100	22.200	42.100	11.100
Lin et al., 2018	LSTM_DBN Network	2.450	1.450	2.100	0.260	1.890
Lu et al., 2018	ANN, SVM, LSSVM and Gaussian process regression	6.433	7.375	5.913	5.500	6.313
Zhang et al., 2018	RBFNN	4.310	5.670	5.520	3.940	4.640
	LSSVM (RBF)	3.960	5.420	2.330	1.690	3.130
Liu et al., 2019	Wavelet Least SVM and Imperialist Competition Algorithm	4.168	0.1684	1.993	0.9675	1.854
This approach	Wavelet-like transform/MLP neural network	5.763	1.831	1.525	2.869	5.069

## Nomenclature

ANFIS	Adaptive Neuro Fuzzy Inference
BPNN	Backpropagation Neural Network
C_2_H_2_	Acetylene Gas
C_2_H_4_	Ethylene Gas
C_2_H_6_	Ethane Gas
CH_4_	Methane Gas
CO	Carbon Monoxide Gas
CO_2_	Carbon Dioxide Gas
Db	Daubechies Wavelets
DGA	Dissolved Gas Analysis
DGC	Dissolved Gas Concentration
DWT	Discrete Wavelet Transform
FIS	Fuzzy Inference System
GE	General Electric
H_2_	Hydrogen Gas
IEC	International Electrotechnical Commission
KMO	Kaiser–Meyer–Olkin
LS-SVM	Vector Machine Least Squares Support
LSTM	Long Short-Term Memory
MAPE	Mean Absolute Percentage Error
MLP	Multi-Layer Perceptron
NARX	Nonlinear Autoregressive Exogenous
PCA	Principal Components Analysis
PTE	Power Transformers
SPCA	Sparse Principal Components Analysis
SVM	Support Vector Machine
TDCG	Total Dissolved Combustible Gas
W-LSSVR	Vector machine least squares support wavelet regression
WT	Wavelet transform
$\psi_{j,k}\left(t\right)$	Mother Wavelet
$\varphi_{m,k}\left(t\right)$	Scaling Function
$H\left(\omega \right)$	Transfer Function of High-Pass Filter
$G\left(\omega \right)$	Transfer Function of Low-Pass Filter
$h_{n}$	High-Pass Coefficients
$\;g_{n}$	Low-Pass Coefficients
$A_{m,k}$	Approximated Profile
$D_{j,k}$	Detail Profile
j,m	Decomposition Level of the Wavelet Transform
